# Antioxidant 1,2,3,4,6-Penta-*O*-galloyl-β-D-glucose Alleviating Apoptosis and Promoting Bone Formation Is Associated with Estrogen Receptors

**DOI:** 10.3390/molecules29215110

**Published:** 2024-10-29

**Authors:** Yongqing Hua, Haili Wang, Tingting Chen, Yeru Zhou, Zhiyuan Chen, Xinyue Zhao, Shaoqin Mo, Hongyun Mao, Miao Li, Linxia Wang, Min Hong

**Affiliations:** 1 Jiangsu Collaborative Innovation Center of Chinese Medicinal Resources Industrialization, National and Local Collaborative Engineering Center of Chinese Medicinal Resources Industrialization and Formulae Innovative Medicine, Nanjing University of Chinese Medicine, Nanjing 210023, China; 2 School of Pharmacy, Nanjing University of Chinese Medicine, Nanjing 210023, China; 3 Jiangsu Key Laboratory for Pharmacology and Safety Evaluation of Chinese Materia Medica, Nanjing University of Chinese Medicine, Nanjing 210023, China; 4 School of Medicine & Holistic Integrative Medicine, Nanjing University of Chinese Medicine, Nanjing 210023, China

**Keywords:** osteoporosis, MC3T3-E1, zebrafish, estrogen receptor α, nuclear factor erythroid 2-related factor 2, reactive oxygen species

## Abstract

1,2,3,4,6-penta-*O*-galloyl-β-D-glucose (PGG) is the main phenolic active ingredient in *Paeoniae Radix Alba*, which is commonly used for the treatment of osteoporosis (OP). PGG is a potent natural antioxidant, and its effects on OP remain unknown. This study aimed to investigate the effects of PGG on promoting bone formation and explore its estrogen receptor (ER)-related mechanisms. A hydrogen peroxide-induced osteoblast apoptosis model was established in MC3T3-E1 cells. The effects of PGG were assessed using 3-(4,5-dimethylthiazol-2-yl)-2,5-diphenyltetrazolium bromide (MTT) assay, flow cytometry, alkaline phosphatase (ALP) staining, RT-qPCR, and Western blot methods. Furthermore, a prednisolone-induced zebrafish OP model was employed to study the effects in vivo. ER inhibitors and molecular docking methods were used further to investigate the interactions between PGG and ERs. The results showed that PGG significantly enhanced cell viability and decreased cell apoptosis by restoring mitochondrial function, attenuating reactive oxygen species levels, decreasing the mitochondrial membrane potential, and enhancing ATP production. PGG enhanced ALP expression and activity and elevated osteogenic differentiation. PGG also promoted bone formation in the zebrafish model, and these effects were reversed by ICI182780. These results provide evidence that the effects of PGG in alleviating apoptosis and promoting bone formation may depend on ERs. As such, PGG is considered a valuable candidate for treating OP.

## 1. Introduction

The 1,2,3,4,6-pentyl-*O*-galloyl-β-D-glucose (PGG) is a naturally available hydrolyzable tannin that belongs to the polyphenol family. A variety of foods and herbs, such as *Juglans mandshurica*, mango, *Toona sinensis*, *Galla rhois, Glycyrrhiza uralensis*, and *Paeonia lactiflora*, commonly contain PGG [1]. Its unique structure with five gallic acyl groups makes PGG one of the most potent antioxidants in tannins, which widely occur in fruits and plants [1,2]. The antioxidant PGG is reported to protect against cardiovascular diseases [3], diabetes mellitus [4], tumors [5,6], etc. PGG is one of the signature components of *Paeoniae Radix Alba*, in which it has the highest concentration of all polyphenolic compounds [7,8]. *Paeoniae Radix Alba*, the peeled and dried root of *Paeonia lactiflora* Pall., is one of the most frequently employed Chinese herbal medicines for treating osteoporosis (OP) [9]; it is included in more than one-third of Traditional Chinese Medicine (TCM) prescriptions for OP [10,11]. However, the exact effects of PGG, the main component of *Paeoniae Radix Alba*, on OP remain unclear.

OP is a chronic disease that impacts the entire skeletal system, reducing bone density and deteriorating the bone’s microscopic structure, resulting in increased bone fragility and risk of fractures [12]. OP is associated with increases in bone resorption, which are regulated by osteoclasts (OCs), and decreases in bone formation, which are mediated by osteoblasts (OBs). OBs are derived from mesenchymal stem cells that are drawn to the bone surface and undergo differentiation into matrix-producing OBs, which subsequently undergo mineralization, resulting in bone formation [13]. Defects in OB function, characterized by heightened OB apoptosis and hindered osteogenic differentiation, are responsible for human bone metabolism diseases such as OP [14]. Suppressing programmed cell death in OBs and enhancing osteogenic differentiation are promising therapeutic strategies for ameliorating OP.

Accumulating evidence has demonstrated that excessive oxidative stress (OS) in OBs contributes to the development of OP [15]. OS derives from an imbalance between oxidation and antioxidation, leading to an excessive accumulation of reactive oxygen species (ROS) [16]. Mitochondria are considered the primary origin of ROS and the principal sites of ROS-induced damage [17]. Excessive ROS generation leads to mitochondrial dysfunction, as evidenced by a significant decrease in membrane potential (MMP) and ATP production. Oxidative stress accelerates OB apoptosis and inhibits OBs’ differentiation [18]. Nuclear transcription factor erythroid-2-related factor 2 (Nrf2) is essential for protecting cells from OS by activating antioxidant genes. This effect is mainly achieved through Nrf2 nuclear translocation [19,20]. Estrogen deficiency, a crucial factor for OP, can lead to increasing OS levels in bone tissue [15,21]. Estrogen increases bone mass by promoting OB proliferation and differentiation [22,23]. The classical estrogen receptors (ERs), ERα and ERβ, are mostly present in the nucleus and act as transcriptional activators in the cell. As novel membrane ERs, GPER (G protein-coupled estrogen receptor) and ER-α36 were both proven to mediate many important functions during bone formation. PGG’s potential to enhance bone formation via its antioxidant properties and association with estrogen receptors requires further investigation.

Currently, the strategy for treating OP involves inhibiting osteoclast activity with anti-resorptive drugs such as bisphosphonate or promoting bone formation with anabolic agents such as the parathyroid hormone. These approaches come with significant side effects [24,25], and more efficacious and safe drugs are still lacking. Exploring the lead compounds derived from herbal medicine may be a valuable drug discovery strategy for OP treatment. Emerging studies have confirmed that polyphenols play an essential role in stimulating bone formation and mineralization and in maintaining OB survival, and these effects are partly due to their antioxidative properties [26]. PGG is a well-known polyphenol that has been found to have antioxidant properties and a significant superoxide molecule-scavenging ability [27]. The H_2_O_2_-induced inhibition of the osteoblast differentiation model in MC3T3-E1 cells is frequently used to investigate whether drugs repair oxidative stress-induced osteoblast damage [18,28]. The bone development and metabolism of zebrafish are similar to homo sapiens, making it a commonly used model in bone research [29]. Therefore, the H_2_O_2_-induced MC3T3-E1 osteogenic differentiation inhibition model and zebrafish bone formation inhibition model were used in this study. This research aimed to examine the influence of PGG in promoting bone formation and inhibiting apoptosis under oxidative stress as well as its association with estrogen receptors.

## 2. Results

### 2.1. PGG Protects MC3T3-E1 Cells Against H_2_O_2_-Induced Apoptosis

**Figure 1:**
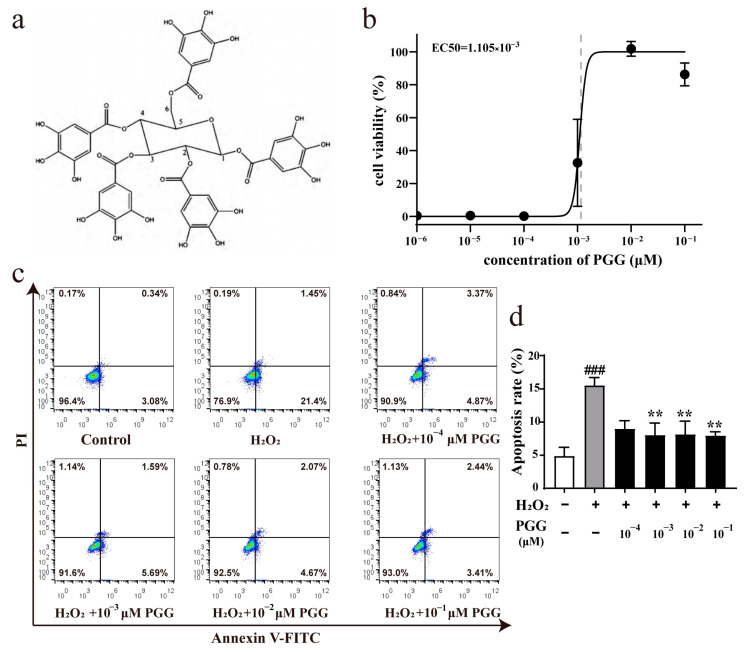
Effects of PGG on H_2_O_2_-induced cell viability decline and apoptosis. (**a**) Chemical structure of PGG. (**b**) Dose–response cell viability curve for PGG. Cells were pretreated with or without H_2_O_2_ (400 μM) for 4 h and then incubated with or without PGG for 24 h. Cell viability was determined via MTT assay. Dose–response curve was quantified based on cell viability by designating control group as 100% and model group as 0% (*n* = 6 per group). (**c**) Cellular apoptosis was assayed using annexin V-FITC and PI counterstaining and analyzed with flow cytometry. Lower-right quadrant cells (Annexin V+/PI−) are early apoptotic cells, and upper-right quadrant cells (Annexin V+/PI+) are late apoptotic cells. Apoptosis rate is calculated via the sum of early and late apoptotic cells. (**d**) Apoptosis rate quantification (*n* = 3 per group; ^###^ *p* < 0.001 vs. control group; ** *p* < 0.01 vs. the H_2_O_2_ group).

To investigate the protective effect of PGG (Figure 1a) against H_2_O_2_-induced OS, MC3T3-E1 cells were pretreated with H_2_O_2_ (400 μM) for 4 h and were then incubated with PGG for 24 h. After this, cell viability and apoptosis were evaluated. The 3-(4,5-dimethylthiazol-2-yl)-2,5-diphenyltetrazolium bromide (MTT) assay results showed that MC3T3-E1 cells exposed to H_2_O_2_ alone displayed markedly decreased vitality, whereas PGG substantially attenuated this at a minimum concentration of 1 × 10^−3^ μM (Figure S1). The dose–response cell viability curves for PGG are shown in Figure 1b. The half-maximal effective concentration (EC_50_) of PGG for mitigating the reduced cell viability induced by H_2_O_2_ is 1.1 × 10^−3^ μM (Figure 1b). H_2_O_2_ significantly increases apoptosis [30]. The results of flow cytometry showed that H_2_O_2_-induced apoptosis was effectively inhibited by PGG (Figure 1c,d). These data illustrate the protective effect of PGG on MC3T3-E1 cells against H_2_O_2_-induced apoptosis. The anti-apoptotic effect of PGG reflects its role in improving cell viability.

### 2.2. PGG Treatment Stimulates MC3T3-E1 Cell Differentiation

**Figure 2:**
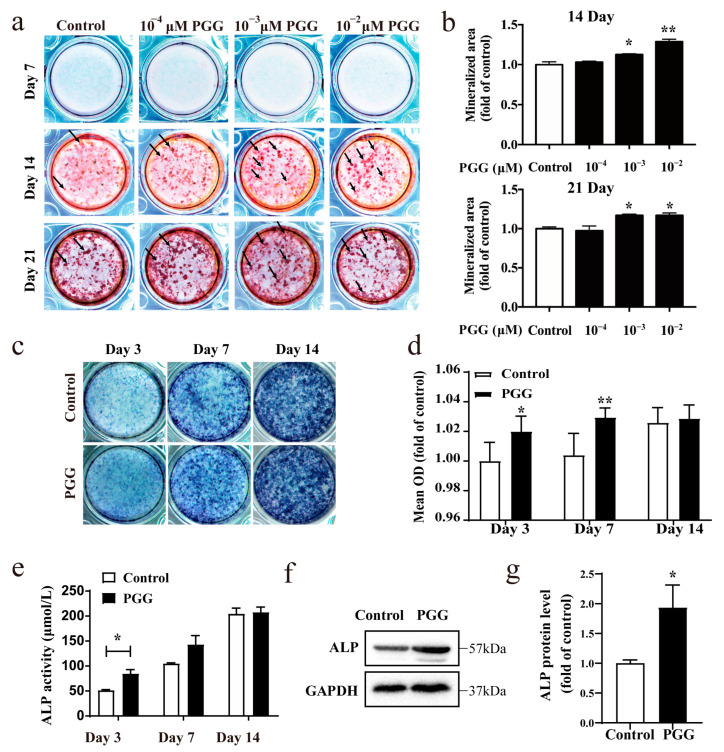
Effect of PGG on osteogenic differentiation in MC3T3-E1 cells. (**a**) ARS staining of MC3T3-E1 cells after 7, 14, and 21 days in osteogenic medium supplemented with 1 × 10^−4^ to 1 × 10^−2^ μM PGG or without PGG. ARS staining appears in red, indicating calcium deposition. (**b**) Quantification of ARS staining to indicate mineralized area (*n* = 3 per group; * *p* < 0.05 and ** *p* < 0.01 vs. control group). (**c**) ALP staining of MC3T3-E1 cells after 3, 7, and 14 days in osteogenic medium supplemented with 1 × 10^−3^ μM PGG. ALP staining appears in blue, indicating the level of ALP activity. (**d**) Quantification of mean optical density of ALP staining (*n* = 5 per group; * *p* < 0.05 and ** *p* < 0.01 vs. control group). (**e**) ALP activity detection (*n* = 3 per group; * *p* < 0.05 vs. the control group). (**f**) ALP protein expression detected via Western blot. (**g**) ALP protein expression quantification in MC3T3-E1 cells (*n* = 3 per group; * *p* < 0.05 vs. control group).

To determine the effects of PGG on OB differentiation, MC3T3-E1 cells were stimulated to differentiate into OBs using osteoblast induction medium (OIM), with or without PGG (1 × 10^−4^ μM, 1 × 10^−3^ μM, 1 × 10^−2^ μM). The calcified nodules were evaluated via Alizarin Red S (ARS) staining on days 7, 14, and 21. On the seventh day, there were no calcium nodules observed in any group. On day 14, a significantly greater number of calcified nodules were observed in both the 1 × 10^−3^ μM PGG and 1 × 10^−2^ μM PGG treatment groups compared to the control group (Figure 2a,b). On day 21, there were large amounts of calcified nodules in the PGG treatment group (Figure 2a,b). ARS staining results indicate that PGG ultimately increases OB differentiation.

ALP promotes hydroxyapatite formation and is regarded as a marker of early OB differentiation, playing a key role in bone formation [31]; ALP activity during the early phase (days 3–7) changes rapidly, and cells exhibit sensitivity to treatment [32,33]. Therefore, we performed an ALP staining and activity assay on MC3T3-E1 cells. PGG notably increased the ALP staining on days 3 and 7 (Figure 2c,d). As shown in Figure 2e, PGG significantly enhanced ALP activity on day 3 compared to the control group. By day 14, osteogenic differentiation reached maturation and ALP activity became saturated, making PGG’s effects on this indicator less apparent or similar to the control group. In addition, qRT-PCR results showed that PGG increased the mRNA levels of the early and late OB differentiation markers, ALP and OCN (Figure S2). Meanwhile, as detected via Western blotting, the protein expression of ALP also increased after PGG treatment (Figure 2f,g). These results indicate that PGG enhances ALP expression and activity and promotes osteogenic differentiation in MC3T3-E1 cells.

### 2.3. PGG Alleviates H_2_O_2_-Induced Mitochondrial Dysfunction in MC3T3-E1 Cells

**Figure 3:**
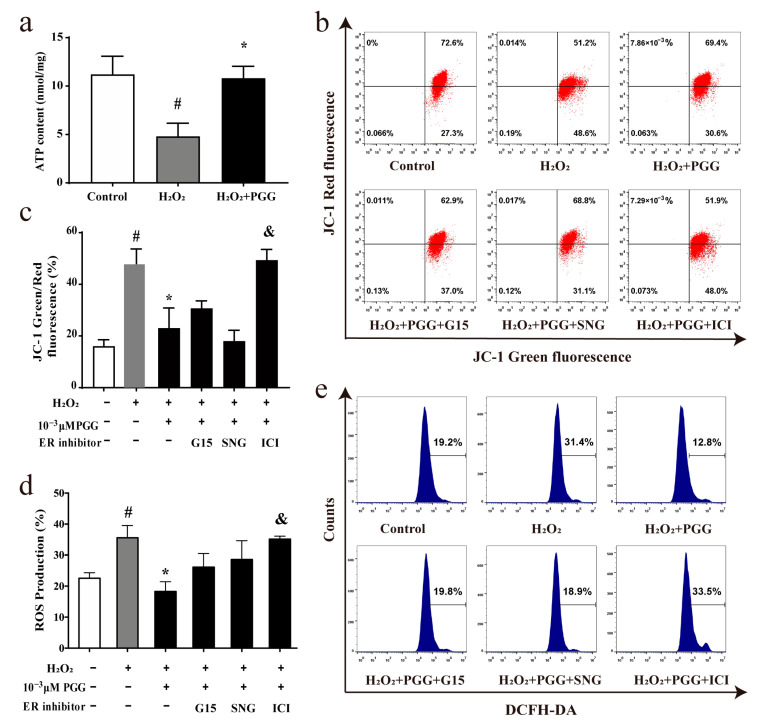
Protective effect of PGG against H_2_O_2_-induced mitochondrial dysfunction in MC3T3-E1 cells and its relation to ERs. (**a**) The impact of PGG on H_2_O_2_-induced mitochondrial ATP production in MC3T3-E1 cells (*n* = 3 per group; ^#^ *p* < 0.05 vs. the control group; * *p* < 0.05 vs. the H_2_O_2_ group). (**b**) The MMP level of MC3T3-E1 cells after co-culture with PGG and ER inhibitors detected by flow cytometry. (**c**) Quantification of MMP level (*n* = 3 per group; ^#^ *p* < 0.05 vs. the control group; * *p* < 0.05 vs. the H_2_O_2_ group; ^&^ *p* < 0.05 vs. the PGG group). (**d**) Quantification of ROS production (*n* = 3 per group; ^#^ *p* < 0.05 vs. the control group; * *p* < 0.05 vs. the H_2_O_2_ group; ^&^ *p* < 0.05 vs. the PGG group). (**e**) The effect of ER inhibitors on the PGG-induced decrease in ROS levels produced by H_2_O_2_ in MC3T3-E1 cells was detected by flow cytometry.

Mitochondria are the central regulatory hubs of cellular energy metabolism and signal regulation, supporting cell growth and differentiation and regulating the process of apoptosis [34]. Under OS, elevated ROS induce mitochondrial damage and dysfunction, leading to an increase in MMP and a decrease in ATP production [35]. To determine whether PGG inhibited MC3T3-E1 cell apoptosis by repairing mitochondrial dysfunction, we employed ATP kit assays, MMP assays, and ROS level measurements to detect mitochondria performance. Cells treated with H_2_O_2_ showed a decrease in ATP production. PGG (1 × 10^−3^ μM) treatment notably restored cellular ATP production, as shown in Figure 3a. Flow cytometry and fluorescence images showed that H_2_O_2_ treatment increased the MMP. The impact of H_2_O_2_ was prevented by PGG treatment (Figure 3b,c). Flow cytometric analysis revealed that PGG decreased the intracellular ROS level (Figure 3d,e), and these findings suggest that PGG restored mitochondrial function under OS.

### 2.4. ICI182780 Reverses the Protective Effect of PGG Against H_2_O_2_-Induced Mitochondrial Dysfunction in MC3T3-E1 Cells

To further investigate whether the protective effects of PGG in H_2_O_2_-treated MC3T3-E1 cells were associated with ERs, G15 [36,37] (a specific antagonist of GPR30, 100 nM), SNG1153 [36] (a specific antagonist of ER-α36, 100 nM), and ICI182780 [36] (nuclear ER antagonist, 100 nM) were used to cotreat MC3T3-E1 cells with PGG for 24 h after H_2_O_2_ treatment. The results showed that ICI182780 significantly reversed the PGG-caused MMP decrease in MC3T3-E1 cells, while no significant G15 and SNG1153 effects were observed (Figure 3b,c). Compared with the PGG treatment group, ICI182780 significantly reversed the ROS scavenging effect of PGG (Figure 3d,e). These findings suggest that PGG restores mitochondrial function under OS on MC3T3-E1 cells, which is associated with nuclear ERs.

### 2.5. ICI182780 Reverses the Promotion of Nrf2 Nuclear Translocation in H_2_O_2_-Treated MC3T3-E1 Cells Caused by PGG

**Figure 4:**
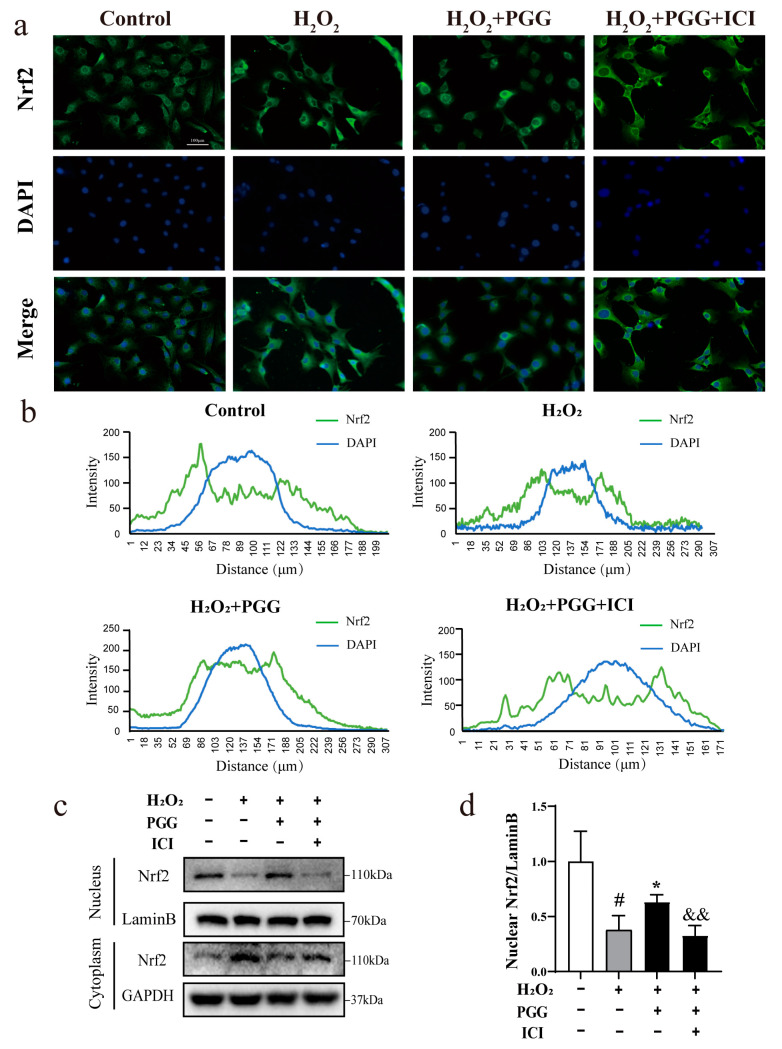
Effect of PGG on Nrf2 nuclear translocation in H_2_O_2_-treated MC3T3-E1 cells and its relation to nuclear ERs. (**a**) Immunofluorescence was used to detect Nrf2 nuclear translocation after co-culture with PGG and ER inhibitor. Nrf2 localization was determined with an anti-Nrf2 antibody (green, fluorescent staining). Nuclear location was determined via DAPI staining (blue, fluorescent staining) (100×, scale bar = 100 μm). (**b**) Fluorescence intensity changes in Nrf2 and DAPI along the line were measured and analyzed via the Plot Profile tool in ImageJ. The overlap of the peak fluorescence intensities of Nrf2 and DAPI reflects the extent of Nrf2 nuclear translocation. (**c**) Nrf2 nuclear translocation after co-culture with PGG and ER inhibitor was detected by Western blot. (**d**) Nuclear Nrf2 protein expression quantification in MC3T3-E1 cells (*n* = 3 per group; ^#^ *p* < 0.05 vs. control group; * *p* < 0.05 vs. H_2_O_2_ group; ^&&^ *p* < 0.01 vs. the PGG group).

Nrf2 nuclear translocation is an important cellular mechanism that activates the antioxidant defense system in response to oxidative stress [38]. The effect of PGG on Nrf2 nuclear translocation and the influence of ICI182780 on this process were evaluated via immunofluorescence. Compared with the control group, the H_2_O_2_ group had a small amount of Nrf2 nuclear translocation, in which Nrf2 is mainly distributed in the cytoplasm. Compared with the H_2_O_2_ group, the PGG group showed a reduction in cytoplasmic Nrf2 and a significant increase in Nrf2 nuclear translocation. ICI182780 markedly inhibits PGG’s promoting effect in Nrf2 nuclear translocation (Figure 4a,b). As illustrated in Figure 4c,d, compared to the control group, cells treated with H_2_O_2_ showed a decrease in nuclear Nrf2 expression. Compared to the H_2_O_2_ group, PGG (1 × 10^−3^ μM) treatment notably promoted the expression of nuclear Nrf2. ICI182780 significantly reversed the PGG-initiated increase in Nrf2 nuclear translocation increase in MC3T3-E1 cells. These results support the findings of the immunofluorescence assay, indicating that PGG facilitates the nuclear translocation of Nrf2 and that the progress is associated with nuclear ERs.

### 2.6. ICI182780 Reverses the Anti-Apoptotic Effect of PGG in H_2_O_2_-Treated MC3T3-E1 Cells and Its Promotive Effects on OB Differentiation

**Figure 5:**
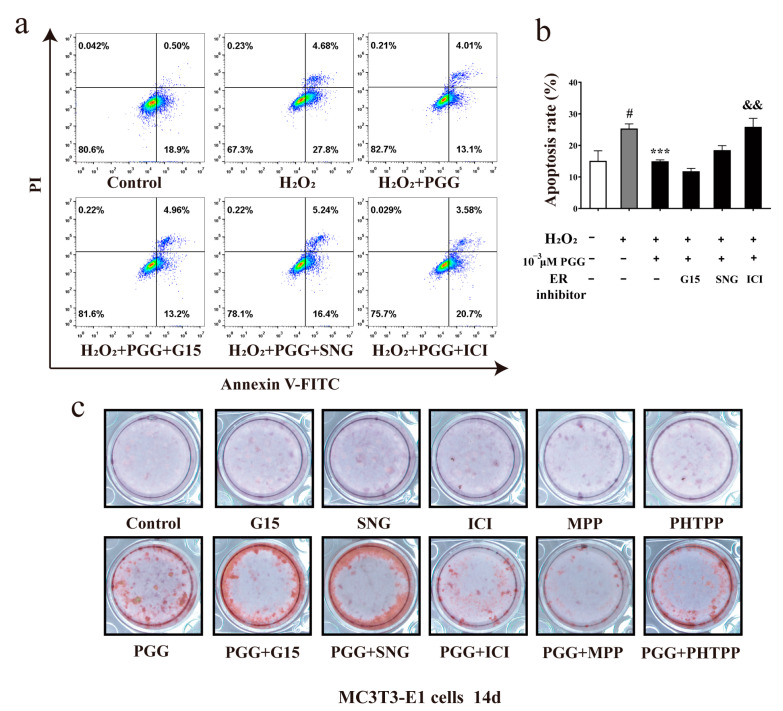
Effect of PGG against H_2_O_2_-induced apoptosis and OB differentiation and its relation with ERs. (**a**) The apoptosis of MC3T3-E1 cells after treatment with PGG and ER inhibitors was detected by flow cytometry. (**b**) Quantification of apoptosis rate (*n* = 3 per group; ^#^ *p* < 0.05 vs. the control group; *** *p* < 0.001 vs. the H_2_O_2_ group; ^&&^ *p* < 0.01 vs. the PGG group). (**c**) ARS staining of MC3T3-E1 cells cultured in OIM for 14 days with PGG and ER inhibitors. ARS staining appears in red, indicating calcium deposition. G15, GPR30 antagonist; SNG1153, ER-α36 antagonist; ICI182780, nuclear ER antagonist; MPP, ERα antagonist; and PHTPP, ERβ antagonist.

To verify whether PGG’s anti-apoptotic and promoting differentiation effects on MC3T3-E1 cells were associated with nuclear ERs, including ERα and ERβ, flow cytometry and ARS staining were employed. The flow cytometry results showed that ICI182780 inhibited PGG’s anti-apoptotic effect (Figure 5a,b). In addition to the previously used inhibitors, MPP (a specific antagonist of ERα) and PHTPP (ERβ antagonist) were used. MC3T3-E1 cells were stimulated to differentiate into OBs using OIM with PGG. The ERα antagonist MPP obviously inhibited the differentiation effect that PGG stimulated in MC3T3-E1 cells. The impact of other ER antagonists on the effect of PGG is not notable (Figure 5c). Therefore, these data indicate that the mechanism responsible for PGG in resisting OB apoptosis and promoting bone formation is associated with ERα.

### 2.7. PGG Promotes Bone Formation In Vivo

**Figure 6:**
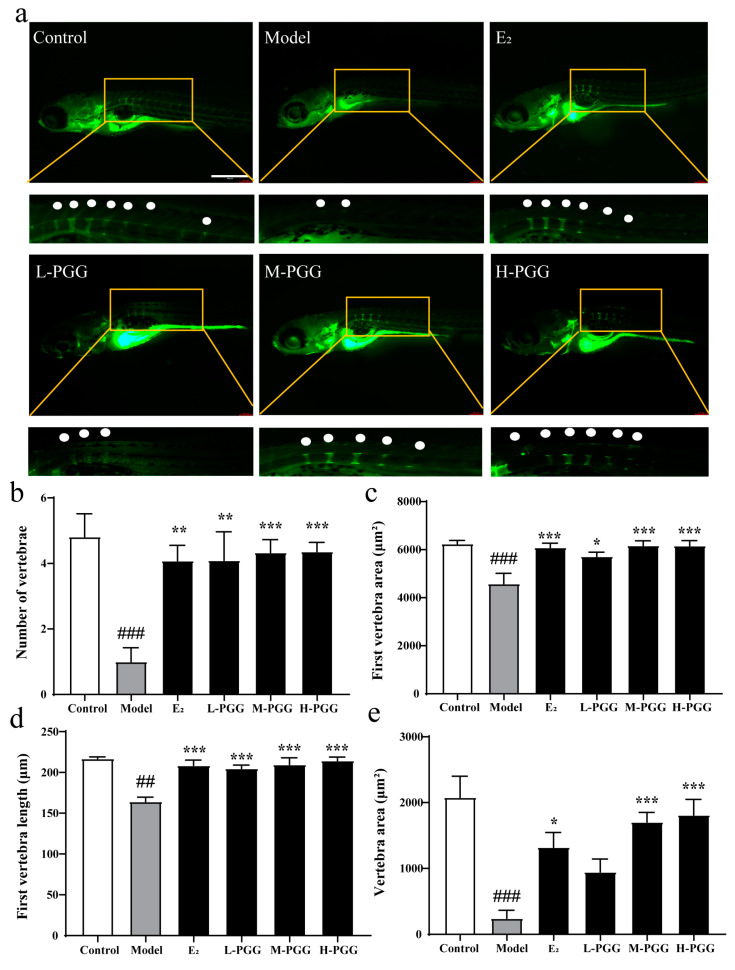
Effect of PGG on zebrafish bone formation inhibiting model. (**a**) Calcein staining of zebrafish. Green represents the Calcein-stained areas, indicating bone tissue. The bone nodes are magnified and marked with white dots. (**b**) Quantification of the number of vertebrae (*n* = 11–12). (**c**) Quantification of the first vertebrae area (*n* = 12). (**d**) Quantification of the first vertebrae length (*n* = 12). (**e**) Quantification of the vertebrae area (*n* = 12) (^##^ *p* < 0.01, ^###^ *p* < 0.001 vs. the control group; * *p* < 0.05, ** *p* < 0.01, *** *p* < 0.001 vs. the model group). Control: blank control group with 0.1% DMSO; model: model group with 10 μM prednisolone and 0.1% DMSO; E_2_: 1 × 10^−2^ μM 17β-estradiol group with 0.1% DMSO and 10 μM prednisolone; L-PGG: low dosage PGG (1 × 10^−4^ μM) with 0.1% DMSO and 10 μM prednisolone; M-PGG: middle dosage PGG (1 × 10^−3^ μM) with 0.1% DMSO and 10 μM prednisolone; H-PGG: high dosage PGG (1 × 10^−2^ μM) with 0.1% DMSO and 10 μM prednisolone.

In this in vitro study, we found that PGG alleviates H_2_O_2_-induced MC3T3-E1 cell cytotoxicity and apoptosis, promoting osteogenic differentiation. To validate the osteoprotective effects of PGG in vivo, we employed a prednisolone-induced zebrafish bone formation inhibiting model, which is widely used in osteoporosis research [39]. As indicated in the calcein staining, compared to the control group, the zebrafish in the model group exhibited a decrease in the number of vertebrae, vertebra area, first vertebra area, and length of the first vertebra. All PGG groups had a significant increase in the number of vertebrae, vertebra area, first vertebra area, and length of the first vertebra compared to the model group (Figure 6a–e). The above results show that PGG has a remarkable effect in promoting bone formation, which is consistent with previous in vitro results.

### 2.8. PGG Exhibits Antioxidative Activity In Vivo

**Figure 7:**
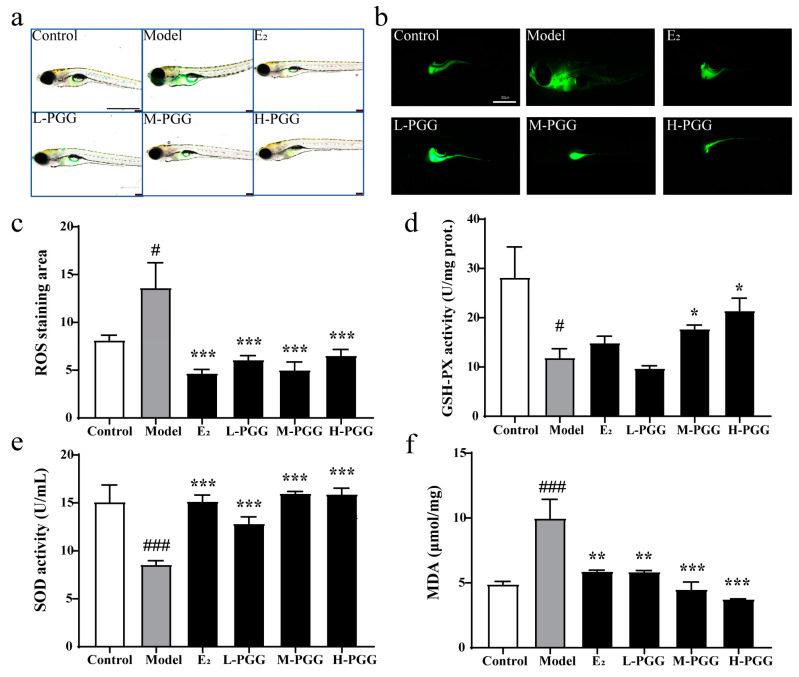
Antioxidant effect of PGG on zebrafish bone formation inhibiting model (**a**) DCFH-DA staining in a bright field was used to observe the ROS level and antioxidative activity effect of PGG on zebrafish. Green fluorescence indicates ROS in the zebrafish. (**b**) DCFH-DA staining in a dark field on zebrafish. (**c**) Quantification of the ROS staining area (*n* = 11–13). (**d**) Quantification of the GSH-PX activity. (**e**) Quantification of the SOD activity. (**f**) Quantification of the MDA level (*n* = 3–5; ^#^ *p* < 0.05, ^###^ *p* < 0.001 vs. the control group; * *p* < 0.05, ** *p* < 0.01, and *** *p* < 0.001 vs. the model group). Control: blank control group with 0.1% DMSO; model: model group with 10 μM prednisolone and 0.1% DMSO; E_2_: 1 × 10^−2^ μM 17β-estradiol group with 0.1% DMSO and 10 μM prednisolone; L-PGG: low dosage PGG (1 × 10^−4^ μM) with 0.1% DMSO and 10 μM prednisolone; M-PGG: middle dosage PGG (1 × 10^−3^ μM) with 0.1% DMSO and 10 μM prednisolone; H-PGG: high dosage PGG (1 × 10^−2^ μM) with 0.1% DMSO and 10 μM prednisolone.

To verify whether the bone-protective effects of PGG are related to its antioxidant properties in vivo, we used DCFH-DA, an ROS fluorescence probe, for observation. The model group exhibited a high ROS level compared with the control group [40], and PGG (1 × 10^−4^ μM, 1 × 10^−3^ μM, 1 × 10^−2^ μM) obviously ameliorated the high ROS level (Figure 7a–c). Under PGG treatment (1 × 10^−3^ μM, 1 × 10^−2^ μM), as shown in Figure 7d, the GSH-PX activity increased significantly (*p* < 0.05). Compared to the model group (Figure 7e), PGG (1 × 10^−4^ μM, 1 × 10^−3^ μM, 1 × 10^−2^ μM) noticeably increased SOD activity (*p* < 0.001). Malondialdehyde (MDA) is one of the most important products produced in the lipid peroxidation process, and it also reflects oxidative stress levels. PGG administration (1 × 10^−4^ μM, 1 × 10^−3^ μM, 1 × 10^−2^ μM) significantly lowered MDA levels (*p* < 0.01, *p* < 0.001), which were raised in the model group (Figure 7f). These data suggest that PGG has notable antioxidant properties, which is consistent with its bone-protective effects.

### 2.9. The Bone-Protective Effects of PGG Probably Interact in Correlation with Nuclear Estrogen Receptors

**Figure 8:**
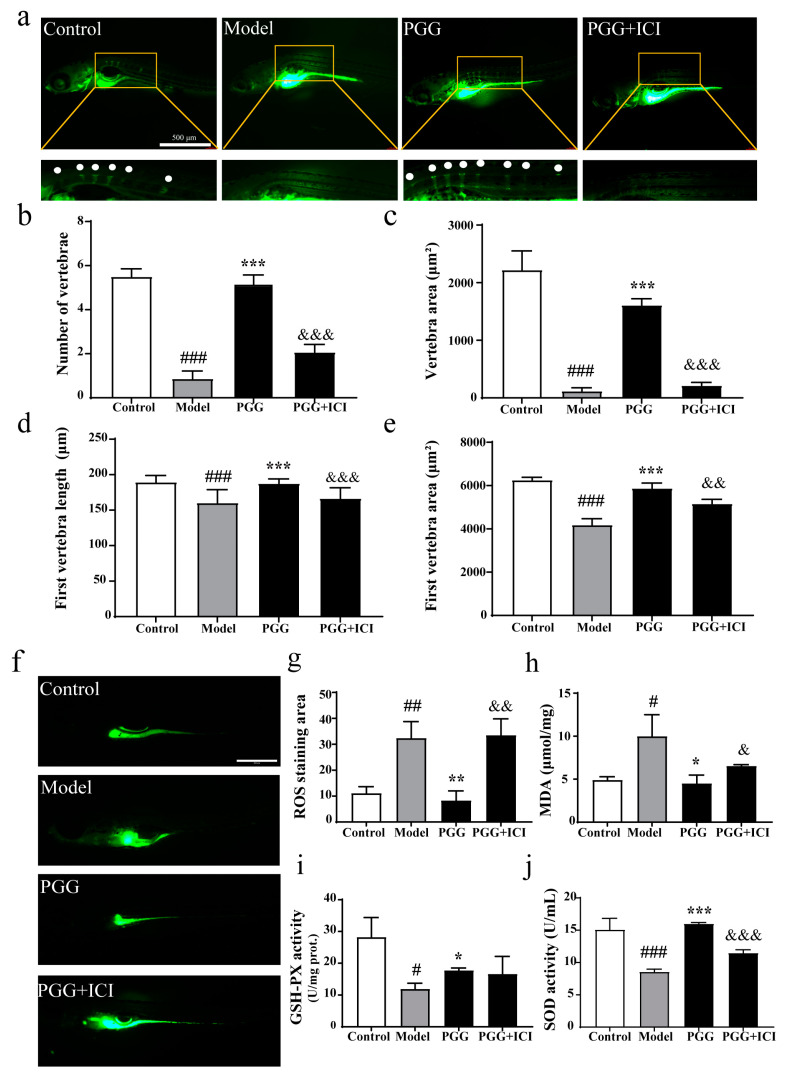
The impact of PGG on the zebrafish bone formation inhibiting model and its correlation with nuclear ERs. (**a**). Calcein staining of zebrafish. Green represents the Calcein-stained areas, indicating bone tissue. The bone nodes are magnified and marked with white dots. (**b**) Quantification of the number of vertebrae (*n* = 14–15). (**c**) Quantification of the vertebrae area (*n* = 13–17). (**d**) Quantification of the first vertebrae length (*n* = 14–16). (**e**) Quantification of the first vertebrae area (*n* = 15–18). (**f**). ROS staining of zebrafish. Green fluorescence indicates ROS in the zebrafish. (**g**) Quantification of ROS staining of zebrafish (*n* = 11–13). (**h**) Quantification of MDA level. (**i**) Quantification of GSH-PX activity level. (**j**) Quantification of SOD activity level (*n* = 3–6). (^#^ *p* < 0.05, ^##^ *p* < 0.01 and ^###^ *p* < 0.001 vs. the control group; * *p* < 0.05, ** *p* < 0.01 and *** *p* < 0.001 vs. the model group; ^&^ *p* < 0.05, ^&&^ *p* < 0.01, and ^&&&^ *p* < 0.001 vs. the PGG group). Control: blank control group with 0.1% DMSO; model: model group with 10 μM prednisolone and 0.1% DMSO; PGG: 1 × 10^−3^ μM PGG with 0.1% DMSO and 10 μM prednisolone; PGG + ICI: 1 × 10^−3^ μM PGG with 0.1% DMSO and 10 μM prednisolone and 100nM ICI182780.

To further demonstrate whether PGG’s antioxidant effect plays a bone-protective role in vivo and whether it is related to nuclear ERs, we used ICI182780, an antagonist of nuclear ERs. As indicated via calcein staining, PGG (1 × 10^−3^ μM) significantly increased the number and area of vertebrae, first vertebra area, and length of the first vertebra compared to the model group. The osteoprotective effects of PGG were reversed upon ICI182780 treatment (Figure 8a–e). These results demonstrate that PGG provides a protective effect on bone and is correlated with nuclear ERs.

Previous in vitro results showed that PGG’s effects on MC3T3-E1 cells are related to its antioxidant properties and nuclear ERs. We conducted further in vivo validation in zebrafish. The findings in Figure 8f–j also matched the in vitro results. As shown in Figure 8f,g, after PGG application, the ROS staining area significantly decreased, compared to the model group (*p* < 0.01). ICI182780 reversed the effect of PGG on ROS reduction. PGG significantly reduced the increased MDA levels in the model group (*p* < 0.05), and ICI182780 reversed this ameliorative effect (Figure 8h). Furthermore, PGG notably increased GSH-PX activity (*p* < 0.5), while the impact of ICI182780 on PGG treatment was not obvious (Figure 8i). In addition, SOD activity levels were increased significantly after PGG treatment (*p* < 0.001) and decreased obviously after ICI182780 treatment (Figure 8j). These results indicate that the antioxidant and bone formation promoting effects of PGG are related to nuclear ERs.

### 2.10. PGG Binds Closely with ERα and Remains Stable

**Figure 9:**
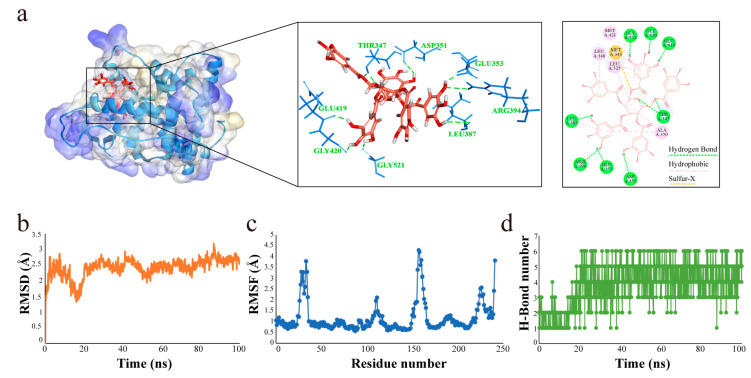
Molecular docking and MD simulation of PGG bound to ERα. (**a**) Molecular docking results for PGG and ERα. (**b**) The backbone RMSD, (**c**) RMSF, and (**d**) H-bond number for the enzyme–ligand complex during the MD simulation.

To further investigate how PGG interacts with nuclear ERs, we carried out the molecular docking of PGG with nuclear ERs (ERα and ERβ). The docking results revealed that the compound PGG entered into the active site of ERα and formed H-bonds with Glu419, Gly420, Gly521, Glu353, Asp351, Leu387, Arg394, and Thr347. The hydrophobic group of the PGG interacted with the lipophilic residues Leu346, Met421, Ala350, and Leu525 (Figure 9a). However, PGG had no affinity for ERβ in this study.

To obtain stable binding conformation and further validate the docking results, a molecular dynamics (MD) simulation was performed for PGG-ERα in an explicit aqueous solution. Overall, the root mean square deviation (RMSD) did not fluctuate significantly, with the RMSD values of all systems converging at 2.5 Å for the 100 ns period (Figure 9b). The majority of the root mean square fluctuation (RMSF) values of the amino acid residues were less than 1.0 Å (Figure 9c). As shown in Figure 9d, most of the H-bond numbers were higher than four. These results indicate that the combination of PGG and ERα was stable.

The binding free energies were calculated using MM-GBSA programs in AMBER 16 (Table 1). Overall, the predicted binding free energy was less than zero, implying the binding interaction between ERα and the compounds could be spontaneous. In particular, PGG had a low binding free energy of −201.40 kcal·mol^−1^, suggesting a stable ligand conformation and potent binding affinity. The binding of PGG was competitive due to higher van der Waals (−114.53 kcal·mol^−1^) and non-polar solvation (−95.46 kcal·mol^−1^) interaction energies. Thus, the compound PGG was an energetically favorable ERα ligand.

## 3. Discussion

This study provides evidence that PGG is a pivotal regulator in promoting OB differentiation and decreasing OBs’ apoptosis under ROS. The mechanism involves the promotion of Nrf2 translocation from the cytoplasm to the nucleus, which prevents ROS production, decreases MMP, improves ATP production, and thereby inhibits OB apoptosis. Furthermore, the impact of PGG on OBs may be related to ERα.

Bone remodeling is a balanced dynamic process based on homeostasis between bone resorption and bone formation [41,42]. This process is initiated by osteoclastic resorption, which erodes a resorption lacuna, allowing OBs to differentiate into osteocytes and fill it. In OP, the main defect is that OBs are unable to refill the resorption lacuna, leading to a net bone loss with each remodeling event [43]. Therefore, promoting bone formation is an important therapeutic strategy against OP. ALP is known to be a critical and early regulator of osteogenesis, and OCN is often defined as a late marker for bone formation [44]. Polyphenols have been found to stimulate bone formation. Diao [45] found that polyphenols (S3) elevated ALP activity in OBs and enhanced mechanical properties under simulated microgravity. Similarly, in our study, we found that polyphenol PGG elevated ALP activity and enhanced the mRNA levels of ALP and OCN, promoting the production of calcified nodules. Furthermore, bone formation was found to be promoted by PGG in vivo in zebrafish. Such evidence suggests that PGG is a substance with potential anti-osteoporosis effects.

Mitochondria are unique and irreplaceable organelles in eukaryotic cells that mediate calcium homeostasis and cell growth and death [46]. Excessive ROS can lead to increased MMP and ATP depletion via cyclophilin D activation, which promotes the sustained opening of the mitochondria permeability transition pore complex [47]. Furthermore, excessive ROS can activate the cytosolic protein BAX, which is thought to participate in mitochondria-mediated apoptosis by forming pores on the mitochondria [48]. Nrf2 is essential for regulating mitochondrial function; in its activated state, it provides cytoprotection against numerous pathologies of chronic diseases [19]. It suppresses ROS generation via translocation into the nucleus and then induces the expression of antioxidant enzymes such as nicotinamide adenine dinucleotide phosphate and heme oxygenase 1 [49]. In this study, we found that PGG decreased OB apoptosis and restored mitochondrial function by scavenging ROS and elevating MMP and ATP production. Moreover, PGG’s amelioration of OB mitochondrial function under OS was associated with promoting Nrf2 nuclear translocation. Therefore, the anti-apoptotic effect of PGG on OBs may involve restoring mitochondrial function.

Estrogen is a female steroid hormone and an essential regulator of various biological processes, such as the development and maintenance of reproductive organs and bone maintenance [50,51]. Studies confirmed that, due to its antioxidant properties, estrogen decreases OS and attenuates the prevalence of OB apoptosis [52]. Estrogen typically binds to ERs, including ERα and Erβ in the cytoplasm. These ERs mediate the transcription of various estrogen-target genes by either binding to estrogen response elements or interacting with other DNA binding proteins, subsequently modulating gene transcription [53]. Researchers produced mice with a deactivated ERα gene that exhibit more severe OP symptoms in estrogen-target tissues compared to mice with a deactivated ERβ gene. These symptoms include a heightened bone resorption and trabecular bone mass [54]. It was postulated that the major physiological actions of estrogen are mediated through ERα, which appears to be its primary receptor [55]. Membrane estrogen receptors, such as G protein-coupled receptor (GPR30) and ER-α36, are involved in regulating bone metabolism. Research has also shown that GPR30 activation could lead to the activation of the Erk/MAPK signaling pathway and, subsequently, could stimulate proliferation and differentiation [56]. ER-α36, a variant of ERα, mediated anti-apoptotic signaling in OBs [57]. Estrogen may interfere with osteoblasts through different mechanisms. At the same time, the relationship between the membrane and nuclear estrogen receptors has not been fully elucidated. Our present study showed that ER antagonists suppressed the osteoprotective effect of PGG. In contrast, this effect was not significantly eliminated by membrane estrogen receptor antagonists. Based on these findings, we surmise that nuclear ERs, rather than membrane ERs, mediate PGG’s osteoprotective effect.

Nrf2 is a key transcription factor that plays an important role in responding to oxidative stress. It is typically inhibited by Keap1 (Kelch-like ECH-associated protein 1) [58]. However, when cells are subjected to oxidative stress, Nrf2 is released from Keap1 and transferred to the nucleus to activate the expression of a series of antioxidant genes [59]. These genes include genes encoding antioxidant enzymes (such as superoxide dismutase and glutathione peroxidase) [60]. ERα plays an important role in antioxidation and influences the activation of the Nrf2-antioxidative function. It may directly or indirectly interact with Nrf2, regulating the expression of antioxidant-related proteins [61]. Chu et al. [62] found that silibinin has protective effects on pancreatic β-cells via Nrf2-antioxidative signaling pathways, which are ERα-dependent. Icariin, a flavonoid that has anti-aging effects, was proven to protect against Sertoli cell injury, which is impaired by aging, via the activation of the ERα/Nrf2 signaling pathway [63]. ERα was verified to act as an upstream regulator of Nrf2. The ERα antagonist effectively inhibited the silychristin-activated Nrf2-HO-1/SOD2 antioxidative pathway [64]. In our study, we found that PGG could promote Nrf2 nuclear translocation and exert an antioxidant effect, thus promoting bone formation. This effect was abrogated by the ERα antagonist, indicating that the effect of PGG is related to ERα. However, further study is needed to accurately understand the role of ERα in Nrf2 interaction.

PGG plays different roles in different tissues and diseases. Regarding tumor cells, PGG has exhibited great potential in treating and preventing cancer, such as nasopharyngeal cancer [5], non-small lung cancer, colorectal cancer, pancreatic cancer, prostate cancer, as well as hepatoma [65], and cachexia [66]. PGG was found to block the MCF-7 cell cycle [67], and the inhibition of ERα by lysosome-dependent depletion was considered to be related to this effect [68]. PGG was also found to have effects on acute lung injury [69], hypertension [70], diabetic nephropathy [71], and inflammation [72]. More specifically, in these studies, PGG activates the Nrf2 pathway and reduces oxidative stress, which results in anti-aging and -apoptotic effects. In this study, we found that PGG provided bone protection in an MC3T3-E1 cell apoptosis model by promoting Nrf2 nuclear translocation, which is the key step in activating the Nrf2 signaling pathway [73]. In addition, we verified that these effects are associated with ERα and ROS scavenging. The tissue-selective effects demonstrated by PGG were similar to those of selective estrogen receptor modulators (SERMs). SERMs are compounds that exhibit tissue-specific ER agonist or antagonist activity. They have been utilized to treat various diseases including breast cancer [74], OP [75], and vulvovaginal atrophy. To date, SERMs’ mechanisms have been briefly explained. The agonistic or antagonistic effects of a SERM in a specific target tissue are influenced by multiple factors, including the differential expression of estrogen receptor subtypes, ERα and ERβ; the conformational changes in the estrogen receptor upon ligand binding; the presence and activity of coregulators; and the tissue-specific cellular environment [76]. Accordingly, we hypothesized that the bone-protective mechanisms of PGG may relate to it acting as a potential SERM. However, its mechanism needs to be studied in further depth.

Estrogen deficiency is the major cause of postmenopausal osteoporosis (PMOP) [15,77]. After menopause, decreased estrogen leads to an imbalance in bone metabolism, which presents as inhibited osteoblast differentiation and activated osteoclast formation [78]. The rapid decrease in estrogen can also cause various diseases during perimenopause, such as cognitive decline, emotional abnormalities, obesity, and cardiovascular disease [79]. By interacting with estrogen receptors, PGG could potentially promote osteoblast activity, improve osteogenic differentiation, and promote bone formation. Given that the bone remodeling process is mediated by both osteoblastic and osteoclastic activities, a comprehensive exploration of the broader effects of PGG on bone homeostasis would include not only effects on osteoblasts but also effects on osteoclastogenesis. PGG may also have effects on osteoclasts through ERs. Our research could not directly prove this effect and mechanism, and further experiments are needed for validation. PGG may have the potential to become a new option for the treatment of perimenopause-related diseases.

## 4. Materials and Methods

### 4.1. Chemicals

PGG was obtained from Shanghai Yuanye Bio-Technology Co., Ltd. (Shanghai, China). The GPR30 antagonist G15 was bought from Cayman Chemical Company (Wuhan, China). The ER-α36 antagonist SNG1153 and the ER antagonist ICI182780 were purchased from Med Chem Express Company (Princeton, NJ, USA).

### 4.2. Cell Culture and Treatment

The pre-osteoblast MC3T3-E1 cells were obtained from the cell library of the Shanghai Chinese Academy of Sciences (Shanghai, China). MC3T3-E1 cells were cultured in α-MEM supplemented with 10% fetal bovine serum (FBS; Gibco, Grand Island, New York, NY, USA) and 1% penicillin-streptomycin (P/S, TransGen Biotech, Beijing, China) at 37 °C with 5% CO_2_ in a humidified atmosphere. The medium was replenished every 2 days.

The final concentrations of the compounds were as follows: H_2_O_2_ (400 μM), the GPR30 antagonist G15 (100 nM), the ERα 36 antagonist SNG1153 (100 nM), the ER antagonist ICI182780 (100 nM), the ERα antagonist MPP (100 nM), the ERβ antagonist PHTPP (100 nM) [36,56], and PGG (1 × 10^−6^, 1 × 10^−5^, 1 × 10^−4^, 1 × 10^−3^, 1 × 10^−2^, and 1 × 10^−1^ μM). The control group was given a complete culture medium containing 0.1% DMSO. The H_2_O_2_ group was given a complete culture medium containing 0.1% DMSO with 400 μM H_2_O_2_. The PGG group was given the corresponding concentration of PGG. G15, SNG1153, ICI182780, MPP, and PHTPP were administered individually at a concentration of 100nM; and the G15 + PGG group, SNG1153 + PGG group, ICI182780 + PGG group, MPP + PGG group, and PHTPP + PGG group were administered PGG at a concentration of 1 × 10⁻^3^ μM, followed by the corresponding concentrations of estrogen receptor antagonists. The drugs were dissolved in a complete culture medium containing 0.1% DMSO.

### 4.3. Cell Viability

MTT assays were performed to evaluate cell proliferation and viability [36]. Briefly, MC3T3-E1 cells were seeded into 96-well plates (1 × 10^4^ cells/well). The cells were then cultured in growth media for a duration of 12 h at a temperature of 37 °C. Afterward, the cells were then exposed to a concentration of 400 μM H_2_O_2_ for 4 h and different concentrations of PGG for 24 h. Then, the MC3T3-E1 cells were washed twice with D-Hanks and incubated in 200 μL/well, serum-free medium supplemented with 20 μL MTT solution (5 mg/mL; BIO FROXX, Einhausen, Hesse, Germany) at 37 °C. After 4 h, the supernatant was removed and the formazan crystals were dissolved via incubation with 150 µL/well DMSO for 10 min. The absorbance of the plates was then read at 490 nm in a microplate reader (BioTek, SyneRgy2; BioTek, Winooski, VT, USA).

### 4.4. Flow Cytometry of Cell Apoptosis

MC3T3-E1 cells were cultured in wells (60 mm × 35 mm), with a cell density of 3 × 10^5^ cells per well, for 24 h. Subsequently, the cells were treated with H_2_O_2_ with or without different PGG concentrations for the indicated periods [36]. The cells were gathered and then reconstituted in 500 μL of binding buffer containing 5 μL of Annexin VFITC and 5 μL of PI, following the manufacturer’s protocol, for 20 min. Subsequently, the cells were rinsed twice with D-Hanks and then bathed with ice for 30 min. All samples were analyzed via flow cytometry (Beckman Coulter, Brea, CA, USA). The apoptosis rate is calculated from the sum of early and late apoptotic cells.

### 4.5. ROS Measurement

Intracellular ROS were detected [80] using an ROS assay kit (Beyotime, Shanghai, China). The cells were collected and then washed with D-Hanks solution. After that, the cells were then placed in a 10 μM DCFH-DA solution and incubated at 37 °C for 20 min. The samples were analyzed via flow cytometry (Beckman Coulter).

### 4.6. Intracellular ATP Production Measurement [81]

ATP production was detected using an ATP assay kit (Beyotime) following the manufacturer’s protocol. Briefly, MC3T3-E1 cells (3 × 10^5^ cells) were lysed using ATP detection lysate. After adding an ATP luciferase detection solution, bioluminescence measurements were obtained using a microplate reader. Additionally, the protein concentration was quantified utilizing the BCA protein assay kit (Beyotime). The emitted light exhibited a linear relationship with ATP concentration and was quantified using a microplate reader (Glomax, Promega, Madison, WI, USA).

### 4.7. Measurement of MMP

The 5,5′,6,6′-terachloro-1,1′,3,3′-tetraethylbenzimidazol-carbocyanine iodide (JC-1), lipophilic cationic fluorescent dye, was employed to identify alterations in the MMP. MC3T3-E1 cells (3 × 10^5^ cells) were cultured in 6-well plates. The MTT measurements were conducted using the Mitochondrial Membrane Potential Detection Kit (Beyotime) according to the manufacturer’s protocol. Briefly, cells were collected and incubated with the MMP-sensitive fluorescent dye JC-1 (10 μg/mL) at 37 °C for 20 min. Next, two washes in the D-Hanks solution followed. All the samples were subjected to flow cytometry (BD Accuri C6, Parkland, FL, USA).

### 4.8. Alizarin Red S Staining

MC3T3-E1 cells (5 × 10^4^ cells/well) were seeded into 12-well plates in the OIM, consisting of α-MEM with 10 mM β-glycerophosphate, 50 mg/mL L-ascorbic acid, 10% FBS, 1% P/S, and 10 nM dexamethasone (Sigma, St. Louis, MO, USA) [36]. Cells were incubated in OIM for 7, 14, and 21 days, fixed with 4% paraformaldehyde at room temperature for 10 min, washed twice with phosphate-buffered saline (PBS), and then stained at room temperature with ARS stain (Servicebio, Wuhan, China) for 5 min. The stained cells were washed with ddH_2_O and put into an oven at 60 °C for 30 min. Mineralized matrix formation was observed under a stereomicroscope (ZEISS, Stemi 2000-C, Zeiss, Jena, Germany).

### 4.9. Alkaline Phosphatase Staining

MC3T3-E1 cells (5 × 10^4^ cells/well) were seeded into 12-well plates. After incubation in OIM for 3, 7, and 14 days, the cells were fixed with 4% paraformaldehyde at room temperature for 10 min, washed twice with PBS, and then stained at room temperature with the BCIP/NBT Alkaline Phosphatase Color Development Kit (Beyotime) for 30 min. The stained cells were washed with ddH_2_O and placed in an oven at 60 °C for 30 min. Mineralized matrix formation was observed under a stereomicroscope (ZEISS, Stemi 2000-C, Carl Zeiss Microscopy GmbH, Jena, Germany).

### 4.10. Alkaline Phosphatase Activity Assay

MC3T3-E1 cells (5 × 10^4^ cells/well) were seeded into 12-well plates. After incubation in OIM for 3, 7, and 14 days, the medium was discarded, the cells were washed twice with PBS, and RIPA buffer (Beyotime) was added. The lysed cells were centrifuged at 4 °C at 13,362 rcf for 10 min. The Alkaline Phosphatase Assay Kit (Beyotime) was used for ALP activity analysis. Firstly, a tube of chromogenic substrate was dissolved in 2.5 mL of detection buffer. Secondly, 50 μL of lysate and 50 μL of chromogenic substrate were added into a 96-well plate and then incubated for 30 min at 37 °C in the dark. Finally, 100 μL of reaction stop solution was added, and absorbance was determined at a wavelength of 405 nm using a microplate reader (Bio-Tek, SyneRgy2, Winooski, VT, USA).

### 4.11. The qRT-PCR

Total RNA was isolated and purified using the RNA Isolater^®^ Total RNA Extraction Reagent (Vazyme Biotech, Nanjing, China). RNA concentrations were quantified utilizing the Synergy2 Multi-mode Microplate Reader. Complementary DNA (cDNA) was synthesized from 1 mg of total RNA using the HiScript^®^ II Q RT SuperMix for qPCR (+g DNA wiper) (Vazyme Biotech, Nanjing, China). Real-time PCR was performed utilizing the ABI 7500 Sequencing Detection System (Applied Biosystems, Foster City, CA, USA) in conjunction with the AceQ^®^ qPCR SYBR^®^ Green Master Mix (Vazyme Biotech, Q111-02). Samples were analyzed utilizing a 7500 Fast Real-Time PCR System (Applied Biosystems). PCR amplification was performed on 2 μL samples of each cDNA using the following specific primers: ALP forward (F): 5′-TGCCTTGCCTGTATCTGGAA-3′, reverse (R): 5′-TGGGAATCTGTGCAGTCTGT-3′; Osteocalcin (OCN) F: 5′-CAGCCATGAGTCAAGTCAGC-3′, R: 5′-TGTGGCTGTGAAACTTGTGG-3′; and GAPDH F: 5′-GGTTGTCTCCTGCGACTTCA-3′, R: 5′-TGGTCCAGGGTTTCTTACTCC-3′.

### 4.12. Immunofluorescence [82]

MC3T3-E1 cells were fixed in 4% paraformaldehyde for 10 min, washed twice with PBS, then permeated with 0.1% Triton X-100, and blocked with 1.5% BSA for 1 h. The cells were next incubated with primary antibodies (rabbit polyclonal anti-Nrf2, 1:50, Proteintech, Chicago, IL, USA) overnight at 4 °C. After washing with PBS, the cells were incubated with secondary antibodies (FITC-tagged goat anti-rabbit, 1:200, Abcam, Cambridge, UK) for 1 h. Cells were then washed with PBS and further incubated with DAPI (1:1000, Beyotime) for 15 min. Fluorescence images were obtained using a live cell-imaging analysis system (ZEISS, Observer.Z1, Jena, Germany). Fluorescence intensity changes in Nrf2 and DAPI along the line were measured and analyzed using the Plot Profile tool in ImageJ. The overlap of the peak fluorescence intensities of Nrf2 and DAPI reflected the extent of Nrf2 nuclear translocation.

### 4.13. Zebrafish Maintenance and Ethics Statement

Adult wild-type AB strain zebrafish, commonly used in bone studies [36,39], were purchased from Eze-Rinka Company (Nanjing, China) and individually housed in a culture system operating on a 14-h light and 10-h dark cycle. All treatments were performed at 28.5 ± 0.5 °C. Zebrafish were maintained in E3 medium (5 mM NaCl, 0.17 mM KCl, 0.33 mM CaCl_2_, 0.33 mM MgSO_4_). The experimentation was performed in the Zebrafish Laboratory (NJUCM, Nanjing, China), conforming to the Technical Requirements for Breeding Laboratory Zebrafish (DB32/T 3979-2021) [83] and with authorization from Nanjing University of Chinese Medicine Laboratory Animal Ethics Committee for zebrafish experimentation (Prot. No. ACU211208, 31 December 2021).

### 4.14. Establishment of a Zebrafish Bone Formation Inhibition Model and Treatments

The zebrafish were placed in a breeding tank at a female/male ratio of 1:2 overnight. The clapboard was taken out the next morning, and embryos were obtained after 1 h under strong light irradiation. Zebrafish embryos were collected after spawning and fertilization; the day of fertilization was 0 dpf (days post fertilization). The 3 dpf larvae were randomly divided into several groups: control group, model group (10 μM prednisolone), E_2_ group (1 × 10^−2^ μM), PGG group (1 × 10^−4^, 1 × 10^−3^, 1 × 10^−2^ μM), and PGG + ICI group (1 × 10^−3^ μM PGG + 100 nM ICI182780). Each group was transferred to six-well plates, with 6 mL of E3 medium per well. From 3 dpf to 5 dpf, the control group was given E3 medium containing 0.1% DMSO, and the model group and each administration group were given 10 μM prednisolone and 0.1% DMSO for modeling. The E_2_ and other administration groups also received respective concentrations of drugs with 10 μM prednisolone in E3 medium. At 9 dpf, calcein staining, DCFH-DA staining, and antioxidant kits were used to assess bone-protective and antioxidation effects. All groups were fed twice a day with paramecia. Each group consisted of 12 zebrafish larvae for studying the effect of PGG on bones, 15 per group for studying the antioxidant effect of PGG, 20 per group for studying the impact of PGG on the zebrafish bone formation inhibiting model and its correlation with nuclear estrogen receptors, and 40 per group for reagent kit testing.

### 4.15. Calcein Staining

The larvae were rinsed clean with fresh water and immersed in 2 mL 0.2% calcein staining solution (pH 7.0) in the dark for 30 min. After staining, the fish were rinsed three times with aquaculture water for 10 min each time to remove the excess unbound calcein from the larvae tissue [84]. Larvae were then euthanized in tricaine methanesulfonate (MS-222) and fixed on glass slides with methyl-cellulose (5%). Bone formation in zebrafish was observed under a LEICA DFC 7000T fluorescence microscope (Leica, Wetzlar, Germany) in the dark. Finally, we used Fiji to count and quantify the area and number of vertebrae.

### 4.16. DCFH-DA Staining

The larvae were rinsed clean with fresh water and immersed in 2 mL 10 μM DCFH-DA staining solution (pH 7.0) in the dark for 20 min [85]. After staining, the fish were rinsed three times with aquaculture water for 10 min each time to remove the excess unbound DCFH-DA from the larvae tissue. The larvae were then euthanized in tricaine methanesulfonate (MS-222) and fixed on glass slides with methyl-cellulose (5%). The ROS staining area in the zebrafish was observed in the dark using a LEICA DFC 7000T fluorescence microscope (Leica, Germany). Finally, we used Fiji to count and quantify the ROS staining area.

### 4.17. Antioxidant Enzyme Activity and Peroxide Detection [86]

The collected zebrafish larvae were prechilled, and PBS was added at a volume of nine times the weight of the larvae (weight (g):volume (mL) = 1:9). The collected zebrafish larvae were homogenized on ice and then centrifuged at 4 °C, 12,000 r/min for 10 min. After the supernatant was collected, glutathione peroxidase (GSH-PX), superoxide dismutase (SOD), and malondialdehyde (MDA) activities were evaluated according to the manufacturer’s protocol. GSH-PX activity was detected using a Glutathione Peroxidase assay kit (Jiancheng, Nanjing, China), SOD activity was detected using a Total Superoxide Dismutase Activity Assay Kit (WST-1 Method) (Elabscience, Wuhan, China), and MDA was detected using the Lipid Peroxidation MDA Assay Kit (Beyotime). Absorbance was measured using a SyneRgy2 Microplate Reader (Bio-Tek, USA).

### 4.18. Nuclear and Cytoplasmic Proteins Extraction

Protein extraction assays were performed according to the protocol of the Nuclear and Cytoplasmic Protein Extraction Kit (Beyotime, Shanghai, China). After MC3T3-E1 cells (5 × 10^4^ cells/well) were collected, 200 μL of cytoplasmic protein extraction reagent A was added to every 20 μL of cells. Cells were vortexed vigorously for 5 s and then lysed on ice for 10–15 min. Next, cytoplasmic protein extraction reagent B was added to the lysed cells. Then, cells were vortexed for 5 s, ice bathed for 1 min, vortexed for 5 s again, and centrifuged at 12,000 rpm for 5 min at 4 °C. The collected supernatant was the cytoplasmic protein. After completely removing the remaining supernatant, 50 μL of nuclear protein extraction reagent was added. We vortexed vigorously for 30 s and repeated the process on ice for 30 min. After being centrifuged at 12,000 rpm for 5 min at 4 °C, the collected supernatant was the nuclear protein.

### 4.19. Western Blot Analysis

MC3T3-E1 cells were harvested and lysed in RIPA buffer after washing twice with PBS. An equivalent quantity of proteins was subjected to 10% SDS-PAGE for separation and subsequently transferred onto polyvinylidene fluoride (PVDF) membranes. After transfer, the membranes were subjected to a blocking procedure utilizing a 5% milk solution. Subsequently, the PVDF membranes were subjected to incubation with primary antibodies: ALP (ab95462, Abcam) and GAPDH (AP0060, Bioworld); Nrf2 (80593-1-RR, Proteintech); LaminB (ab16048, Abcam). Each was diluted at 1:1000. The membranes were incubated for 1 h with secondary antibodies after washing with TBST for 30 min. A digital gel documentation system (ChemiDocTMRS+, BIO-RAD, USA) was used to detect immunoreactive bands.

### 4.20. Molecular Docking [87]

The molecular docking of compounds into the three-dimensional X-ray structures of ERα (PDB ID:3ERT) and ERβ (PDB ID:5TOA) was carried out using the docking module of the Schrödinger Maestro 2019 (Schrödinger, New York, NY, USA). The three-dimensional structure of the compound was constructed using ChemBio 3D Ultra 13.0 software (Chemical Structure Drawing Standard; Cambridge Soft Corp., Cambridge, MA, USA), and it was then energetically minimized using MMFF94 with 5000 iterations and a minimum RMS gradient of 0.05. The protein was prepared using the Protein Preparation Wizard of Schrödinger Maestro 2019. Water molecules were eliminated from the protein and polar hydrogen was added. Receptor grids were generated using the Receptor Grid Generation (Schrödinger). Compound PGG was placed during the molecular docking procedure. The types of interactions of the docked protein with the ligand were analyzed after the completion of molecular docking. Image files were generated using PyMOL 1.1 (Schrödinger).

### 4.21. MD Simulation

A 100 ns MD simulation was carried out; this was performed with the AMBER 16 software package. The reference structures for the protein–ligand complex were taken from the predicted binding structures in molecular docking. The force field parameters for the protein and ligands were generated with Amber ff14SB and the general Amber force field, respectively. The model was solvated in a truncated octahedron box of transferable interaction potential water molecules with a margin distance of 12. Sodium ions were added to neutralize the system. The particle mesh Ewald method [88] was used for long-range electrostatic interactions with a cutoff distance of 10. Next, energy minimizations were performed on the system through 2500 steps of steepest descent followed by 2500 steps of conjugate gradients. After energy minimization, the whole system was gradually heated from 0 K to 300 K for 60 ps under NVT, followed by stimulation for 600 ps at 1 atm under NPT, with harmonic restraints of 2 kcal/(mol·2Å^2^) on the complex. Finally, 100 ns of MD production was performed at 300 K with 1.0 atm pressure. The temperature and pressure were kept constant using a Langevin thermostat and a Langevin barostat separately. All hydrogen atoms were constrained by the SHAKE algorithm and the time step was 2 fs. The resulting trajectories were analyzed with the AMBER16 module CPPTRAJ. The RMSD, RMSF, and hydrogen bonding interactions were analyzed throughout the trajectory using the CPPTRAJ module in AmberTools.

### 4.22. Statistical Analysis

All data were analyzed using GraphPad Prism 7.0 (GraphPad 10.0 Software, La Jolla, CA, USA) and are expressed as means ± SEMs from at least three independent experiments. A one-way analysis of variance (ANOVA) was employed to determine the statistical significance. A *p* < 0.05 was considered a statistically significant difference.

## 5. Conclusions

In summary, PGG intervenes in different processes of oxidative stress to prevent apoptosis, attenuating the level of ROS, decreasing the MMP, and enhancing ATP production. PGG could also significantly enhance ALP and OCN expression, elevate ALP activity, and promote calcification nodule production in MC3T3-E1. Furthermore, PGG promoted bone formation in zebrafish. Our study revealed that, under OS after PGG treatment, the promotion of OB differentiation and inhibition of OB apoptosis are dependent on estrogen receptors. These findings indicate that the antioxidant PGG regulates bone-protective effects in relation to ERα. As a natural polyphenol compound with potential anti-osteoporosis properties, PGG has broad prospects for application in anti-osteoporosis drugs, food supplements, and biomaterials.

## Figures and Tables

**Table 1:** Binding free energy (kcal·mol^−1^) of ligand–protein complexes for the individual energy component contributions.

Contribution	PGG
ΔGVDW a	−114.53 ± 3.95
ΔGele b	−73.08 ± 6.47
ΔGGB c	80.01 ± 4.45
ΔGGA d	−95.46 ± 0.24
ΔGbind e	−201.40 ± 5.13

a Contribution to the free energy of binding from the van der Waals energy. b Contribution to the free energy of binding from the electrostatic energy. c Contribution to the free energy of binding from the polar solvation energies. d Contribution to the free energy of binding from the non-polar solvation energies. e Free energy of binding.

## Data Availability

The data used to support the findings of this study are available from the corresponding author upon request.

## Supplementary Materials

The following supporting information can be downloaded at: https://www.mdpi.com/article/10.3390/molecules29215110/s1, Figure S1. The cells were pretreated with or without H_2_O_2_ (400 μM) for 4 h and then incubated with or without PGG for 24 h. Cell viability was determined via MTT assay (*n* = 6 per group; ^###^ *p* < 0.001 vs. the control group; *** *p* < 0.001 vs. the H_2_O_2_ group); Figure S2. MC3T3-E1 cells were cultured in osteogenic medium supplemented with 10^−3^ μM PGG for 3 days, after which mRNA levels of ALP and OCN were determined. PGG significantly increased the mRNA expression levels of ALP and OCN. (*n* = 3 per group; ** *p* < 0.01 vs. the control group).
