# Supplementary material for: Antioxidant 1,2,3,4,6-Penta-O-galloyl-β-D-glucose Alleviating Apoptosis and Promoting Bone Formation Is Associated with Estrogen Receptors
Source: Molecules. 2024 Oct 29;29(21):5110. doi: 10.3390/molecules29215110 (PMC11547736; doi:10.3390/molecules29215110)
Supplement: Supplementary file 1 [file molecules-29-05110-s001.zip › molecules-3233290-supplementary.pdf]

Supporting information for

Antioxidant 1,2,3,4,6-Penta-*O*-Galloyl-β-D-Glucose Alleviating Apoptosis and Promoting Bone Formation Is Associated with Estrogen Receptors

Yongqing Hua [1, 2, 3*†], Haili Wang [2†], Tingting Chen [2†], Yeru Zhou [4], Zhiyuan Chen [2], Xinyue Zhao [2], Shaoqin Mo [2], Hongyun Mao [2], Miao Li [2], Linxia Wang [2], and Min Hong [1, 2, 3]

[1] Jiangsu Collaborative Innovation Center of Chinese Medicinal Resources Industrialization, and National and Local Collaborative Engineering Center of Chinese Medicinal Resources Industrialization and Formulae Innovative Medicine, Nanjing University of Chinese Medicine, Nanjing 210023, Jiangsu, China;
[2] School of Pharmacy, Nanjing University of Chinese Medicine, Nanjing 210023, Jiangsu, China.
[3] Jiangsu Key Laboratory for Pharmacology and Safety Evaluation of Chinese Materia Medica, Nanjing University of Chinese Medicine, Nanjing 210023, Jiangsu, China
[4] School of Medicine & Holistic Integrative Medicine, Nanjing University of Chinese Medicine, Nanjing, 210023, Jiangsu, China.
*Correspondence: hua_yq@njucm.edu.cn
†These authors have contributed equally to this work and share first authorship.

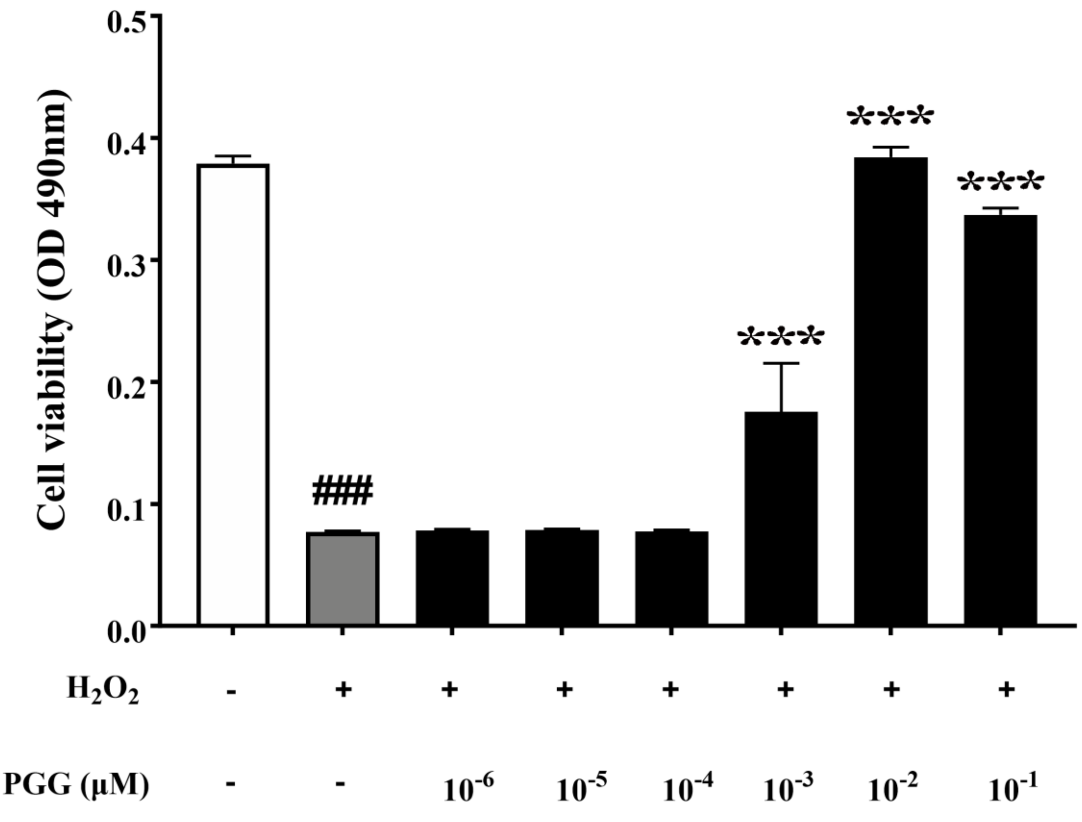

Figure S1. The cells were pretreated with or without H_2O_2 (400 μM) for 4 h and then incubated with or without PGG for 24 h. Cell viability was determined via MTT assay (n = 6 per group; ### P < 0.001 vs. the control group; *** P < 0.001 vs. the H_2O_2

group).

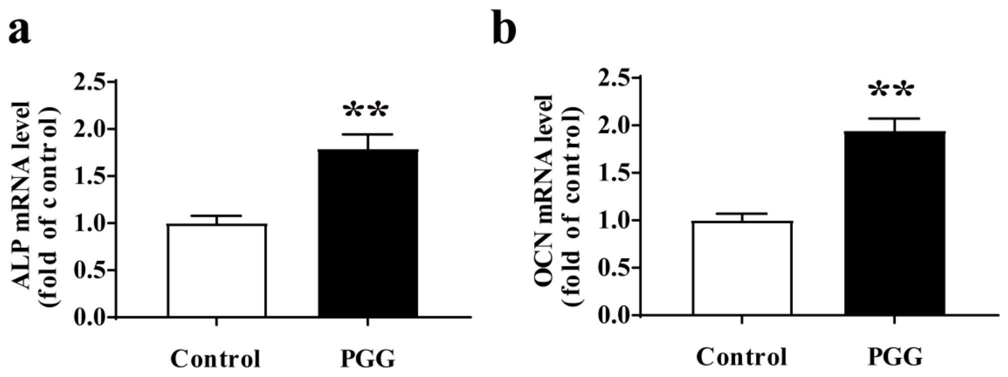

Figure S2. MC3T3-E1 cells were cultured in osteogenic medium supplemented with 10^{-3} μM PGG for 3 days, after which mRNA levels of ALP and OCN were determined. PGG significantly increased the mRNA expression levels of ALP and OCN. (n = 3 per group; ** P < 0.01 vs. the control group).
